# Scalable Synthesis of High-Density Ultrafine Spherical Silver Powders

**DOI:** 10.3390/ma19102010

**Published:** 2026-05-12

**Authors:** Xi He, Jiangyong Pei, Xiaocai He, Ruidong Xu

**Affiliations:** 1 State Key Laboratory of Complex Nonferrous Metal Resources Clean Utilization, Faculty of Metallurgical and Energy Engineering, Kunming University of Science and Technology, Kunming 650093, China; hexi1@stu.kust.edu.cn; 2 School of Metallurgy, Northeastern University, Shenyang 110819, China; peijiangyong@mails.neu.edu.cn; 3 National Key Laboratory of Nonferrous Metal Reinforced Metallurgy New Technology, Nonferrous Metals Research Institute Co., Ltd., Aluminum Corporation of China, Kunming 650021, China; xc_he269@chinalco.com.cn

**Keywords:** photovoltaic silver paste, spherical silver powder, liquid-phase reduction technique, ascorbic acid

## Abstract

Ultrafine spherical Ag powders with narrow particle size distribution, high tap density, and limited agglomeration are important conductive fillers for advanced photovoltaic paste formulation. Current liquid-phase reduction scale-up is limited by uncontrolled nucleation, secondary agglomeration, and precursor passivation. This study investigates a process-integrated synthesis chain from precursor preparation to pilot-scale powder production from precursor preparation to kilogram-scale production. A flow-field-enhanced dissolution process (70–80 °C, 30–40% HNO_3_) alleviates silver ingot passivation, while a multi-stage NaOH spray system reduces NO*_x_* emissions to 186 mg/m^3^, meeting GB31573-2015 standards. Ascorbic acid kinetically decouples nucleation and growth per the LaMer model. Molecular dynamics simulations and RDF analysis reveal a synergistic dispersion mechanism involving PVP and gum arabic. A purpose-built 20 L pilot reactor with optimized fluid dynamics and high-pressure cleaning eliminates supersaturation heterogeneity. Subsequent ethanol displacement and supersonic jet milling yield 1 kg-scale powder with D50 = 1.90 µm, tap density = 6.0 g/mL, specific surface area = 0.6 m^2^/g, and LOI (538 °C) = 0.98%. The obtained powder shows powder-level characteristics relevant to subsequent photovoltaic paste formulation, rather than direct device-level validation.

## 1. Introduction

The imperative global energy transition and the pursuit of carbon neutrality are predicated upon the accelerated maturation, cost reduction, and terawatt-scale deployment of advanced photovoltaic (PV) technologies. As conventional p-type Passivated Emitter and Rear Cells (PERC) approach their theoretical Auger recombination limit, high-efficiency n-type architectures have decisively emerged as the industrial mainstream. Specifically, Tunnel Oxide Passivated Contact (TOPCon) and Silicon Heterojunction (SHJ/HJT) configurations achieve superior photoelectric conversion efficiencies by radically suppressing bipolar recombination and extending minority carrier lifetimes. However, this advanced architectural topology mandates double-sided metallization, precipitating a substantial surge in the consumption of specialized photovoltaic-grade silver pastes [1,2,3,4]. Industrial metrics indicate that while PERCs consume ~10 mg/W of silver, TOPCon architectures require ~13 mg/W, and low-temperature cured SHJ modules demand up to 22 mg/W.

To optimize the levelized cost of electricity (LCOE) and mitigate supply chain volatility, module manufacturers are aggressively advancing silver-thrifting strategies. Paramount among these are zero-busbar (0BB) interconnection technologies and ultra-fine-line screen printing, which drive front-side grid line widths well below 10 μm. Furthermore, the integration of Laser-Enhanced Contact Optimization (LECO) in TOPCon fabrication facilitates Current-Fired Contact (CFC) structures, demanding precisely tailored interactions between the silver powder and glass frit to penetrate the silicon nitride (SiN_X_) anti-reflective coating without compromising the underlying emitter. These advanced metallization techniques impose uncompromising physical, rheological, and chemical specifications on the conductive filler [3,5,6,7]. To ensure flawless thixotropic flow through high-mesh screens and ultra-dense packing during rapid thermal firing (or low-temperature curing), the primary silver powder must exhibit perfect dispersibility, high sphericity, maximal tap density, and an exceptionally low residual carbon profile [8,9,10,11,12]_._

The industrial synthesis of ultrafine spherical silver powder predominantly relies on liquid-phase chemical reduction. Unlike physical methods, such as plasma evaporation, PVD, or high-energy ball milling, which suffer from exorbitant energy costs and broad particle size distributions, liquid-phase reduction offers scalable throughput and atomic-level control over crystallite morphology under mild thermodynamic conditions [13,14,15,16,17,18]. Nevertheless, the transition from laboratory-scale precision to industrial-volume production exposes several interconnected and severe technological bottleneck [9].

First, the precursor stage presents a critical chokepoint. The dissolution of bulk silver ingots into high-purity silver nitrate is hampered by deep surface passivation, wherein a dense layer of poorly soluble silver oxides impedes reaction kinetics and generates environmentally problematic nitrogen oxide (NO*_x_*) exhaust. Second, volumetric scale-up introduces spatiotemporal variations in local supersaturation, leading to overlapping nucleation and growth kinetics that broaden the particle size distribution and yield non-spherical morphologies. Third, within the high-ionic-strength precursor media, the Debye–Hückel screening effect collapses the electrical double layer surrounding nascent nanoparticles, rendering DLVO electrostatic repulsion ineffective and triggering severe secondary hard agglomeration driven by van der Waals forces. Fourth, to counteract agglomeration, high-molecular-weight polymeric surfactants are required to provide steric hindrance. However, this often results in excessive carbon entrapment, leading to elevated Loss-on-Ignition (LOI) values. During rapid thermal firing (750–850 °C), this residual organic matter pyrolyzes into an insulating carbonaceous barrier, disrupting glass frit etching and preventing optimal Ag-Si metallurgical fusion. Finally, the classic “engineering amplification effect” threatens monodispersity, as macroscopic mixing times in large-volume reactors exceed the characteristic timescale of the chemical reaction, generating chaotic concentration gradients.

Addressing these trans-disciplinary challenges requires a holistic approach spanning physical chemistry, interfacial mechanics, and fluid dynamics. The theoretical foundation for monodispersity is governed by the LaMer nucleation model, which dictates that a brief burst of homogeneous nucleation must be followed by a sharp decline in monomer supersaturation to transition exclusively to diffusion-controlled growth [19,20,21,22]. Regarding colloidal stability, single-component dispersant systems exhibit inherent structural limitations. Polyvinylpyrrolidone (PVP), while effective in directing isotropic growth via Lewis coordination with high-energy Ag facets, lacks sufficient hydrodynamic thickness to prevent shear-induced agglomeration in turbulent reactor. Conversely, natural macromolecules like gum arabic (AG), a highly branched arabinogalactan-protein complex, provide a robust hydrodynamic retardation network but lack the specific anchoring groups necessary for permanent passivation of the silver surface, often leading to Ostwald ripening and polyhedral particle formation [23,24,25,26,27,28,29].

The novelty of this work does not lie in proposing a new Ag^+^ reduction reaction, but in integrating precursor dissolution, reduction-kinetics regulation, composite dispersant-assisted morphology control, post-treatment, and kilogram-scale verification into a coherent powder-synthesis route. We first establish an eco-friendly precursor synthesis protocol targeting NO*_x_* suppression and ingot passivation elimination. Utilizing ascorbic acid as a kinetically mild reducing agent, we map the thermodynamic windows (pH, temperature, stoichiometry) required to strictly adhere to LaMer burst kinetics. Through comprehensive molecular dynamics (MD) simulations, we elucidate the atomic-level synergistic “coordination passivation–steric passivation–steric” of a novel PVP/AG composite dispersant system. Crucially, we translate these optimized parameters to a 20 L kilogram-scale pilot environment, implementing specific fluid-dynamic reactor modifications and sequential post-treatments (anhydrous ethanol displacement and supersonic jet-milling) to circumvent the classic scale-up effect. To maintain focus on the primary breakthroughs, intermediate optimization data, including qualitative reducing agent screening and XRD validation of tail-gas byproducts, are systematically provided in the Supplementary Information (SI). The resulting silver powder comprehensively satisfies the extreme purity, high-density packing, and rheological mandates of next-generation n-type high-efficiency photovoltaic cells.

Compared with previous studies mainly focused on laboratory-scale morphology control, this work emphasizes process continuity and scale-up transferability, including precursor preparation, NO*_x_* absorption, pilot-scale reaction control, and post-treatment deagglomeration.

## 2. Experimental

### 2.1. Materials

High-purity metallic silver ingots (4N purity, >99.99%) were used as the primary starting material for precursor synthesis. Silver nitrate (AgNO_3_, >99.8%), L-ascorbic acid (C_6_H_8_O_6_, analytical grade), hydrazine hydrate (N_2_H_4_·H_2_O, analytical grade), formaldehyde solution (HCHO, 37 wt.%, analytical grade), and glucose (C_6_H_12_O_6_, analytical grade) were purchased from Sinopharm Chemical Reagent Co., Ltd. (Shanghai, China).

Polyvinylpyrrolidone K30 (PVP K30), gum arabic (AG), sodium dodecylbenzenesulfonate (SDBS), cetyltrimethylammonium bromide (CTAB), polyethylene glycol 4000 (PEG4000), and Tween 20 were used as dispersants. PVP K30, gum arabic, PEG4000, and Tween 20 were purchased from Shanghai Macklin Biochemical Co., Ltd. (Shanghai, China), while SDBS and CTAB were purchased from Aladdin Biochemical Technology Co., Ltd. (Shanghai, China).

Nitric acid (HNO_3_, analytical grade), aqueous ammonia (NH_3_·H_2_O, analytical grade), sodium hydroxide (NaOH, analytical grade), and anhydrous ethanol (C_2_H_5_OH, analytical grade) were purchased from Sinopharm Chemical Reagent Co., Ltd. (Shanghai, China). Ultrapure water with a resistivity of 18.2 MΩ·cm was used throughout the experiments. All reagents were used as received without further purification unless otherwise stated.

### 2.2. Powder Synthesis

Powder synthesis was carried out in a jacketed reactor equipped with mechanical stirring, temperature control, and controlled precursor feeding. For the laboratory-scale experiments, a 2 L jacketed glass reactor with a working volume of 1.20 L was used. The reactor had an inner diameter of 120 mm and a liquid height of approximately 106 mm during operation. A four-blade pitched-blade turbine impeller with a diameter of 45 mm was positioned 25 mm above the reactor bottom. Four vertical baffles with a width of 12 mm were installed to improve macroscopic mixing and suppress vortex formation. The stirring speed was maintained at 600 rpm during precursor addition and then reduced to 400 rpm during ageing. The reaction temperature was controlled by circulating water through the jacket and monitored using a thermocouple inserted into the liquid phase.

AgNO_3_ solution with a concentration of 2.0 mol/L was used as the silver precursor. L-ascorbic acid solution with a concentration of 0.82 mol/L was used as the reducing agent. For a typical laboratory-scale batch, 465 mL of AgNO_3_ solution was rapidly introduced into 735 mL of ascorbic acid/dispersant solution, corresponding to a theoretical Ag yield of approximately 100 g. PVP K30 and gum arabic were completely dissolved in the ascorbic acid solution before precursor addition. The optimized PVP:AG mass ratio was 1.0:2.0, and the total dispersant dosage was 1.5 wt.% relative to the theoretical Ag mass, corresponding to 0.5 wt.% PVP and 1.0 wt.% gum arabic. The initial pH of the reducing solution was adjusted to 3.0 using dilute HNO_3_ or aqueous NH_3_ before reaction.

A pre-dissolved reverse rapid-addition protocol was adopted. The AgNO_3_ solution was introduced into the ascorbic acid/dispersant solution through a 2 mm feeding tube located approximately 20 mm above the upper edge of the impeller and at a radial distance of about 0.25 times the reactor diameter from the central shaft. The feeding rate was controlled at 100 mL/min for the optimized laboratory-scale experiment, and the addition time was approximately 4.7 min. After addition, the suspension was aged for 30 min under continuous stirring at 40 °C.

For the pilot-scale experiment, the optimized conditions were transferred to a 20 L jacketed stainless-steel reactor with a working volume of 12.0 L. The reactor had an inner diameter of 280 mm and an operating liquid height of approximately 195 mm. The reactor was equipped with a four-blade pitched-blade turbine impeller with a diameter of 100 mm and four vertical baffles with a width of 25 mm. The stirring speed was maintained at 300 rpm during precursor addition and then reduced to 220 rpm during ageing. In a typical kilogram-scale batch, 4.65 L of 2.0 mol/L AgNO_3_ solution was pumped into 7.35 L of 0.82 mol/L ascorbic acid/dispersant solution through a 4 mm feeding tube located near the impeller zone. The AgNO_3_ feeding rate was 1.0 L/min, giving an addition time of approximately 4.7 min. The total precursor amount corresponded to a theoretical Ag yield of approximately 1.0 kg per batch. The main operating parameters for the laboratory- and pilot-scale syntheses are summarized in Table S1.

### 2.3. Post-Treatment

The collected product was washed with ultrapure water (60 °C) until neutral, followed by three displacement washes with anhydrous ethanol to minimize capillary-induced agglomeration. After vacuum drying (40 °C, −0.1 MPa, 12 h), the powder was deagglomerated using a high-pressure flat-type jet mill to reduce weak secondary agglomerates while maintaining the observed particle morphology under the present treatment conditions.

### 2.4. Characterizations and Simulations

The morphology of the Ag powders was observed by scanning electron microscopy (SEM, XL30 ESEM-TMP, FEI/Philips, Hillsboro, OR, USA) at an accelerating voltage of 20 kV. SEM images were used for qualitative observation of particle morphology. Quantitative image analysis of projected circularity, aspect ratio, or true three-dimensional sphericity was not performed in this study.

Particle-size distribution and specific surface area were measured by an external testing institution during the same commissioned characterization procedure. Particle-size distribution was determined by wet laser diffraction using ethanol as the dispersion medium. D10, D50, and D90 represent the particle diameters at cumulative volume fractions of 10%, 50%, and 90%, respectively, and the span was calculated as (D90 − D10)/D50.

The specific surface area was determined independently by nitrogen adsorption using the Brunauer–Emmett–Teller (BET) method. The specific surface area values reported in this work were obtained from the external test report and were not calculated from the particle-size distribution curves.

X-ray diffraction (XRD) was used to identify the crystalline phase composition of the optimized final Ag powder. The XRD pattern was collected using an X-ray diffractometer (D8 Advance, Bruker AXS GmbH, Karlsruhe, Germany) with Cu Kα radiation (λ = 1.5406 Å) at 40 kV and 40 mA. The scanning range was 10–90° with a step size of 0.02°. The obtained pattern was compared with the standard diffraction pattern of metallic Ag, PDF#04-0783. XRD was used only for crystalline-phase identification and cannot exclude trace ionic Ag(I), amorphous residues, or impurities below the detection limit.

Tap density was measured using a tap-density analyzer (YA-ZD-10, Shanghai Leiyun Testing Instrument Manufacturing Co., Ltd., Shanghai, China) at 250 taps/min for 12 min. The measurement was described according to metallic-powder tap-density testing principles consistent with ISO 3953 [30] and ASTM B527 [31].

The specific surface area was determined by nitrogen adsorption using the Brunauer–Emmett–Teller (BET) method with a surface area and porosity analyzer (ASAP 2460, Micromeritics Instrument Corp., Norcross, GA, USA). Before measurement, the powder sample was degassed under vacuum at 80 °C for 6 h to remove physically adsorbed species. Nitrogen adsorption was performed at 77 K, and the specific surface area was calculated using the multipoint BET method.

Loss on ignition (LOI) was measured using a muffle furnace (SX2-4-10, China). The powder sample was heated at 538 °C for 30 min in air, and LOI was calculated from the mass loss before and after heating. LOI was used as a powder-level total mass-loss indicator under a fixed heating condition. Because FTIR/IR, XPS, and TGA analyses were not performed in this study, LOI was not used to identify specific surface-bound organic species, such as PVP or gum arabic.

NO*_x_* concentration at the outlet of the NaOH absorption tower was monitored using an infrared flue-gas analyzer (Gasboard-3000, Cubic-Ruiyi Instrument Co., Ltd., Wuhan, China). The sampling point was located at the outlet of the final absorption stage. The monitoring method was described with reference to HJ 692-2014, which specifies the determination of nitrogen oxides in stationary-source exhaust gas by non-dispersive infrared absorption. The reported maximum value represents the highest outlet NO*_x_* concentration recorded during the Ag dissolution process.

The simulations were designed to compare the relative interfacial adsorption behavior of PVP, gum arabic, and the PVP/gum arabic composite system near the Ag surface. The organic dispersant molecules were described using the COMPASS II force field. A periodic Ag slab model was constructed to represent the exposed Ag surface. The bottom Ag layers were fixed to mimic the bulk lattice, while the upper Ag layers and dispersant molecules were allowed to relax. Dispersant molecules were initially placed above the Ag surface in an aqueous simulation cell.

The simulation procedure consisted of geometry optimization, thermal equilibration, and production molecular dynamics [32,33]. First, the system was optimized using the smart minimization algorithm. Second, the optimized system was equilibrated in the NVT ensemble at 313.15 K for 500 ps. Third, production MD was performed in the NVT ensemble at 313.15 K for 5 ns with a time step of 1 fs. The trajectory from the equilibrated stage was used for structural analysis.

Partial radial distribution functions were calculated between surface Ag atoms and selected heteroatoms in the dispersant molecules. The first-peak position and peak intensity were extracted from the RDF profiles. The average adsorption distance was calculated as the mean vertical distance between the Ag surface plane and the nearest adsorbing heteroatoms. The radius of gyration was calculated from the mass-weighted atomic coordinates of the dispersant molecules. These simulation results were used only to compare relative adsorption proximity and steric-stabilization behavior, rather than to describe electron transfer, Ag(I) reduction, or Ag particle nucleation.

## 3. Results and Discussion

### 3.1. Eco-Friendly Precursor Synthesis and NO_x_ Emission Thermodynamics

The synthesis of high performance, electronic grade silver powder is fundamentally contingent upon the absolute purity of the silver nitrate precursor. Conventional dissolution of bulk metallic silver in concentrated nitric acid encounters a formidable kinetic barrier arising from the rapid formation of a dense, passivating interfacial layer composed predominantly of poorly soluble silver oxides and localized nitrate saturation. This layer severely restricts the outward diffusion of Ag^+^ ions, collapsing the overall dissolution kinetics, exponentially prolonging processing durations, and incurring substantial waste of the high value raw material. To circumvent this kinetic limitation, a precise thermo-chemical matrix was established.

Experimental empirical data (Table S5) confirmed that at lower thermodynamic temperatures (40–50 °C), dissolution times extended beyond eight hours, rendering the process industrially unviable and highly inefficient. Elevating the temperature to the window of 70–80 °C supplied the requisite activation energy to surmount the surface passivation barrier. Simultaneously, maintaining the HNO_3_ mass fraction tightly between 30% and 40% prevented the extreme exothermic runaway characteristic of higher acid concentrations, which otherwise provokes uncontrollable boiling and hazardous reactor overflow. Under these optimized conditions, augmented by flow-field enhanced macroscopic stirring and a staged acid replenishment protocol designed to continuously thin the localized Nernst diffusion layer, the complete and rapid dissolution of 100 g silver batches was reliably achieved within an optimized timeframe of 3–4 h.

Critically, the chemical dissolution of silver in nitric acid inevitably liberates substantial volumes of highly toxic nitrogen oxides (NO*_x_*), constituting a severe ecological and operational hazard [34,35]. To transform this inherently noxious process into a closed-loop green synthesis, a meticulously engineered multi-stage NaOH spray absorption tower was integrated directly into the exhaust manifold. Outlet-gas monitoring using a non-dispersive infrared NO*_x_* analyzer showed that the maximum NO*_x_* concentration after NaOH absorption was 186 mg/m^3^ during the Ag dissolution stage. Under the tested operating conditions, the maximum outlet NO*_x_* concentration after NaOH absorption was 186 mg m^−3^, which was below the emission limit specified in GB 31573-2015. The reproducibility of outlet NO*_x_* concentration and the nitrogen balance of the absorbed NO*_x_* species are summarized in Table S4. This value resides well below the stringent mandates stipulated in the National Emission Standard of Pollutants for Inorganic Chemical Industry (GB31573-2015), thereby ensuring absolute environmental compliance. Furthermore, the liquid effluent from the absorption tower was evaporated to recover solid byproducts. X-ray Photoelectron Spectroscopy and X-ray diffraction (XRD) (detailed in the Supplementary Information Table S7 and Figure S2) confirmed that the recovered solids consisted of high-purity crystalline NaNO_3_ and NaNO_2_, with residual silver content suppressed to a mere 0.004%. This achievement successfully closes the loop on precursor synthesis, effectively converting a toxic exhaust liability into high-value commercial byproducts. The main alkaline absorption reactions can be represented by the following simplified stoichiometric equations [36,37,38]:

NO + NO_2_ + 2NaOH → 2NaNO_2_ + H_2_O (1)

2NO_2_ + 2NaOH → NaNO_3_ + NaNO_2_ + H_2_O (2)

These reactions represent the conversion of absorbed NO*_x_* into nitrite and nitrate salts in alkaline solution. It should be noted that practical NO*_x_* absorption involves gas-phase equilibria, gas–liquid mass transfer, and multiple liquid-phase reactions; therefore, Equations (1) and (2) are used here only as simplified stoichiometric descriptions.

### 3.2. Reduction Kinetics and LaMer Nucleation

**Figure 1:**
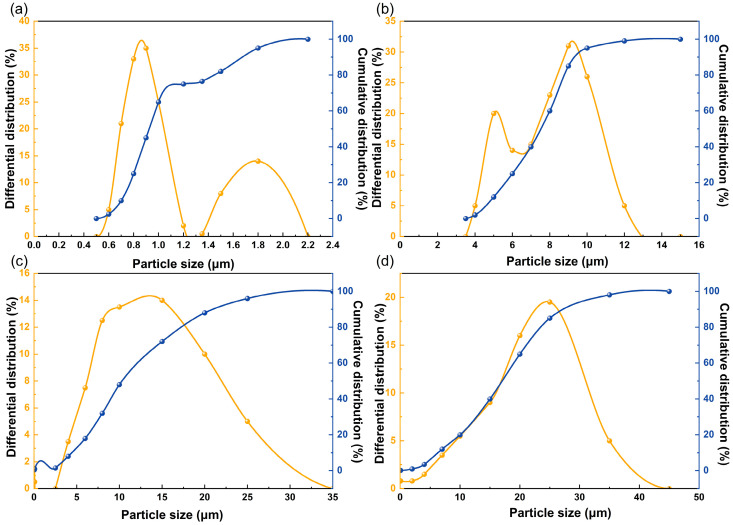
Particle size distributions of Ag powders prepared using different reducing agents: (**a**) hydrazine hydrate; (**b**) formaldehyde; (**c**) ascorbic acid; and (**d**) glucose. The comparison was used to evaluate the influence of reduction kinetics on PSD and agglomeration behavior.

The LaMer nucleation model (Figure S3) postulates that the nucleation mode, whether instantaneous or progressive, is dictated by the balance between the monomer generation rate and the supersaturation consumption rate. In silver powder synthesis, the redox potential of the selected reducing agent directly governs the kinetic profile of silver monomer release. Table 1 and Figure 1 summarizes the effects of various reducing agents on the resulting powder morphology and particle dimensions. The powders obtained using different reducing agents were used for preliminary comparison of particle-size distribution and morphology. Their crystalline phase compositions were not individually verified by XRD; therefore, no purity conclusion is made for these screening samples.

Hydrazine hydrate and formaldehyde exhibit relatively low redox potentials, driving extremely rapid electron-transfer kinetics, allowing the reaction system to instantaneously overcome the critical energy barrier required for nucleation. Under these conditions, the monomer generation rate significantly outpaces both monomer integration and surface diffusion. This kinetic imbalance triggers persistent secondary nucleation alongside continuous crystal growth, yielding a bimodal particle size distribution and severe particle agglomeration. Conversely, glucose in weakly acidic media possesses an excessively positive redox potential, providing insufficient thermodynamic driving force for the reduction reaction. Consequently, the system maintains a low nucleation density, and the reaction proceeds predominantly via Ostwald ripening. This pathway causes irregular crystallite coarsening and produces an exceptionally broad particle size distribution.

In stark contrast, ascorbic acid demonstrates optimal performance in regulating crystallite morphology. This superiority is attributed to its moderate reduction kinetics, enabling the local supersaturation to rapidly surpass the critical nucleation threshold and subsequently undergo a sharp decline. Consequently, nucleation and growth become temporally decoupled, fulfilling a crucial prerequisite for synthesizing monodisperse particles. Furthermore, the extended growth-buffer period facilitates the spontaneous rearrangement of silver atoms onto low-energy surface sites. This atomic rearrangement yields spherical, internally dense, and highly symmetric particles with excellent monodispersity. Based on these thermodynamic and kinetic advantages, ascorbic acid was established as the primary reducing agent for this investigation.

### 3.3. Thermodynamic and Kinetic Control

**Figure 2:**
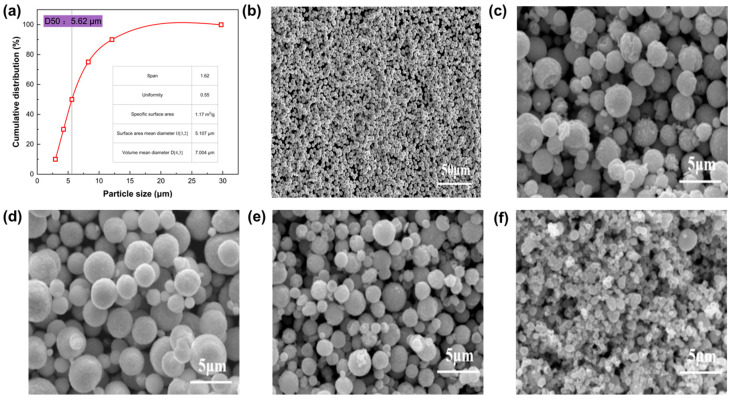
Particle size distribution and microscopic morphology of silver powders synthesised at 40 °C, together with the effect of pH on silver powder morphology: (**a**) particle size distribution at 40 °C; (**b**) microscopic morphology at 40 °C; (**c**) pH = 2.5; (**d**) pH = 3.0; (**e**) pH = 3.5; (**f**) pH = 4.0.

The redox potential of ascorbic acid is inherently pH-dependent. Governed by the Nernst equation, elevated pH increases ascorbic acid dissociation, thereby amplifying the thermodynamic driving force for reductio. At a constant 40 °C, a restricted pH of 2.5 suppresses dissociation and reduction kinetics, driving silver deposition predominantly via epitaxial growth. This induces significant powder coarsening, yielding a D50 of 7.54 μm. Increasing the pH to 3.0 establishes an optimal balance between the reduction driving force and crystallization kinetics. This facilitates independent and uniform crystal growth, producing highly dispersed particles with a D50 of 5.17 μm and a tap density of 4.2 g/mL. However, exceeding pH 3.5 generates an excessively strong reduction potential and uncontrolled supersaturation. This triggers severe primary particle collisions, disrupts internal compactness, and ultimately causes macroscopic agglomeration. The corresponding particle-size distribution and SEM morphology under different pH conditions are shown in Figure 2.

Concurrently, reaction temperature dictates crystallization kinetics, mass-transfer flux, and surface-diffusion capabilities in accordance with the Arrhenius equation. The detailed effects of temperature on the particles size and morphology were listed in Table S8. At 35 °C, insufficient thermal energy deprives silver atoms of the activation energy required for efficient surface rearrangement, significantly compromising crystallite compactness. Conversely, temperatures at or above 45 °C amplify systemic thermal disturbances and internuclear collision frequencies, exacerbating severe agglomeration. Furthermore, such elevated temperatures prematurely arrest crystallite structural evolution before surface-energy minimization can occur, resulting in a marked drop in tap density. Consequently, 40 °C and a pH of 3.0 were established as the optimal thermodynamic parameters to ensure a seamless transition from burst nucleation to diffusion-controlled growth.

### 3.4. Synergistic Interfacial Passivation and Steric Hindrance

In high-concentration liquid-phase precursor systems, the strong electrolyte environment induces a profound Debye–Hückel effect, severely compressing the electrical double layer surrounding colloidal particles. Consequently, classical DLVO electrostatic repulsion is rendered entirely ineffective. Under these conditions, introducing non-ionic polymers to provide robust steric hindrance becomes an essential strategy for maintaining colloidal stability. Table 2 summarizes the molecular-level synergistic mechanisms of the polyvinylpyrrolidone (PVP) and gum arabic (AG) composite dispersant system [23,24,25].

Employed individually, PVP utilizes its lactam rings to coordinate with high-energy silver facets, thereby reducing surface energy and directing isotropic particle growth. However, a solitary PVP monolayer lacks the spatial thickness and mechanical stiffness necessary to prevent shear-induced soft aggregation during mechanical stirring, resulting in a D50 of 9.84 μm. Conversely, the isolated use of the natural macromolecule AG provides a massive, hydrated hydrodynamic retardation network but lacks the specific anchoring groups required for robust chemical passivation. Unpassivated crystallites autonomously minimize their surface energy via dissolution-recrystallization pathways. This triggers severe Ostwald ripening, inflating the D50 to 16.04 μm. Evidently, solitary dispersants fail to achieve simultaneous high monodispersity and maximal tap density.

**Figure 3:**
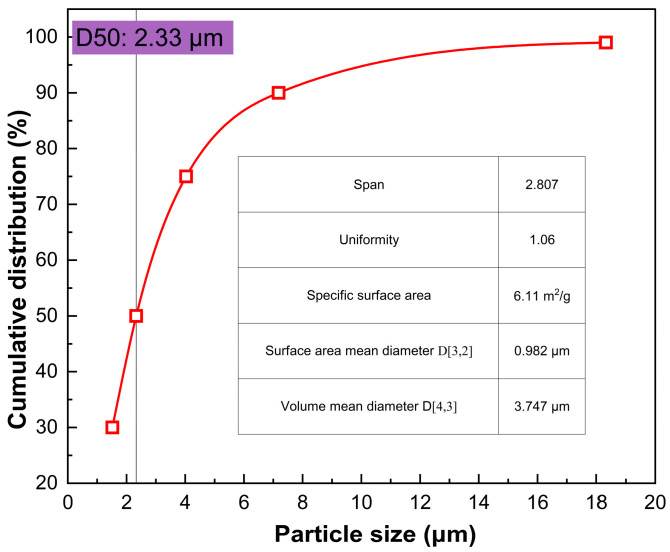
Particle size distribution of Ag powder prepared using the optimized PVP/AG mass ratio of 1.0:2.0.

Blending PVP and AG at a 1.0:2.0 mass ratio unlocks a clear improvement. During the initial reduction phase, the strong coordination capacity of PVP drives its preferential adsorption onto nascent nuclei, establishing a dense passivating inner layer. Subsequently, driven by hydrophobic interactions and macromolecular chain entanglement, high-molecular-weight AG molecules anchor to the exterior of the PVP layer. This self-assembly forms a resilient, hydrated outer shell that delivers additional steric barrier. This hierarchical composite architecture successfully integrates the crystallographic morphology control of PVP with the robust anti-agglomeration dispersion of AG. Driven by this cooperative mechanism, the D50 plunges to 2.33 μm (Figure 3), while the tap density peaks at 4.4 g/mL. These findings support the use of the composite interfacial engineering strategy for scalable, high-density silver powder production.

**Figure 4:**
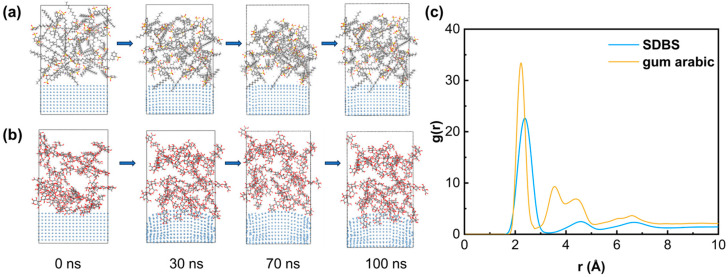
Comparative molecular simulation of interfacial adsorption behavior for a representative small-molecule surfactant and gum arabic. The optimized experimental dispersant system remains PVP/AG. (**a**) Mechanism of the influence of SDBS; (**b**) Mechanism of the influence of AG; (**c**) Radial distribution function (PDF).

To elucidate the dispersant-driven morphological evolution and the formation mechanism of spherical silver particles, molecular dynamics (MD) simulations coupled with radial distribution function (RDF) analyses were performed on sodium dodecylbenzenesulfonate (SDBS) and gum arabic (AG) systems. The comparative structural outcomes are presented in Figure 4.

It should be noted that the optimized experimental dispersant system in this work is PVP/AG. In the molecular simulation, SDBS was included only as a representative small-molecule surfactant for comparison with macromolecular AG adsorption. The purpose of this comparison was not to identify SDBS as the optimized dispersant, but to illustrate the difference between small-molecule adsorption and macromolecular steric stabilization. Therefore, the simulation results are used only as supportive information for understanding interfacial adsorption behavior, while the main experimental conclusion regarding dispersant optimization is based on the PVP/AG system shown in Table 2 and Figure 3.

RDF profiles reveal that AG induces a markedly enhanced short-range order. The initial coordination peak for AG emerges at r = 2.18 Å with an intensity of g(r) = 33.1 and a narrow full-width at half-maximum (FWHM) of 0.21 Å. Conversely, SDBS exhibits a broader and weaker peak shifted to r = 2.32 Å (g(r) = 22.4, FWHM = 0.37 Å). Furthermore, AG displays distinct secondary peaks at 3.85 Å and 4.72 Å, signifying the robust preservation of medium-range structural ordering, whereas the SDBS system presents merely a faint, diffuse profile. These structural discrepancies originate from fundamentally distinct interfacial adsorption behaviors. AG anchors significantly closer to the silver surface (average distance of 3.18 Å versus 4.67 Å for SDBS) and achieves more than double the molecular coverage (18.6 nm^2^ compared to 7.9 nm^2^).

As a natural polysaccharide rich in hydroxyl and carboxyl moieties, AG undergoes extensive multi-site coordination and substantial macromolecular chain contraction upon adsorption. This conformational alteration reduces its radius of gyration by approximately 18.7%, from 2.84 nm to 2.31 nm, thereby forging a dense and homogeneous protective coating. In contrast, SDBS attaches primarily via its sulfonate headgroups with minimal conformational adaptation, yielding an irregularly passivated interface. Consequently, AG more effectively mitigates interfacial roughness, reducing the atomic height dispersion (σ) to 0.41 nm compared to 0.76 nm for SDBS, which facilitates highly isotropic crystalline development.

Upon simulation completion, AG-stabilized silver particles achieve a sphericity of 0.91 and an aspect ratio of 1.08, geometries that closely approximate perfect spheres. Conversely, SDBS yields highly irregular particles with a sphericity of 0.74 and an aspect ratio of 1.37. These molecular insights substantiate that the dense, multi-site, and uniform adsorption of AG effectively suppresses surface energy anisotropy across crystallographic facets. This interfacial passivation is crucial for synthesizing highly spherical silver powders, definitively underscoring the exceptional thermodynamic advantages of AG as an eco-friendly dispersant.

The simulation results should be regarded as supportive evidence for interfacial adsorption behavior rather than direct proof of the complete particle nucleation and growth process.

### 3.5. Stoichiometry and Ageing Kinetics

The molar ratio of ascorbic acid to AgNO_3_ is an important parameter affecting the local reducing environment and the final powder properties. At the theoretical stoichiometric ratio (1.0:2.0), the progressive depletion of the reducing agent induces an insufficient local redox potential. This thermodynamic deficit triggers the microscopic dissolution of newly formed crystallographic facets, culminating in surface roughening. Elevating the molar ratio to 1.3:2.0, providing a 30% theoretical excess, establishes a more stable reducing environment. This environment effectively suppresses reverse dissolution, facilitates the consistent monomeric filling of lattice defects, and densifies the particulate structure. Conversely, a severe excess (e.g., 1.7:2.0) generates an excessively high initial chemical potential, spawning numerous irregular nuclei. Furthermore, surplus organic species undergo may promote residue retention within the lattice interstices, detrimentally elevating the loss-on-ignition (LOI) residue of the final powder product.

**Figure 5:**
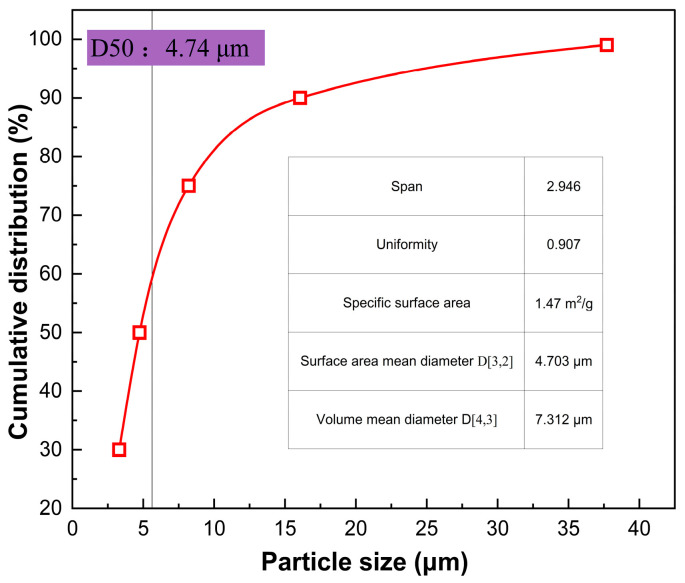
Particle size distribution of silver powder synthesised at a reducing agent to silver nitrate molar ratio of 1.3:2.0.

Beyond precursor stoichiometry, post-reaction ageing critically modulates the surface tension equilibrium and structural relaxation of the particle system. An inadequate ageing duration of 20 min stalls atomic lattice reconfiguration, leaving persistent high-energy surface steps and considerable interparticle frictional forces. Extending the ageing period to 30 min facilitates optimal crystallographic facet rearrangement and surface diffusion. Within this ideal kinetic window, the composite polymeric protective layer undergoes uniform structural relaxation, maximizing the macroscopic packing density governed by the steric barrier. Conversely, prolonged ageing (>40 min) subjects the system to sustained mechanical shear, which compromises the integrity of the peripheral protective layer and may lead to excessive interfacial polymer adsorption. Consequently, severe particle agglomeration ensues, and the 538 °C LOI metrics exceed acceptable thresholds. Therefore, a precursor molar ratio of 1.3:2.0 combined with a 30 min ageing duration was selected as the optimized condition in this study because it provided a favorable balance among particle size distribution, tap density, and LOI. The particle-size distribution obtained under the optimized ascorbic acid/AgNO_3_ molar ratio of 1.3:2.0 is shown in Figure 5.

### 3.6. Post-Treatment and Deagglomeration

For Ag powders used in conductive pastes, post-treatment and deagglomeration are important because residual surface organics, particle agglomeration, specific surface area, and tap density can affect packing, paste formulation, sintering behavior, and final conductivity [37,38]. Inadequate removal of polymeric and inorganic residues during wet-chemical synthesis leads to persistent surface contamination. During the rapid thermal firing (750–850 °C) of N-type solar cells, these species pyrolyze into an amorphous carbon barrier. This insulating phase severely impedes metallurgical fusion between adjacent silver particles and degrades electrode contact resistance. To eradicate this barrier, we implemented an anhydrous ethanol deep solvent-displacement protocol following conventional hot deionized water repulping. Ethanol displacement may help reduce drying-induced agglomeration by lowering capillary forces during solvent removal. The LOI value measured at 538 °C for 30 min was below 1.0%, indicating relatively low total mass loss under the specified test condition. However, because FTIR/IR and TGA analyses were not performed, this LOI result is not used to assign the mass loss to specific surface-bound macromolecules or to claim complete removal of PVP or gum arabic.

Nevertheless, solvent displacement alone cannot entirely avert the soft agglomeration induced by hydrogen bonding during drying, necessitating a secondary mechanical dispersion step. We deployed a high-pressure flat-type jet mill to induce intense aerodynamic shearing, effectively disassembling residual physical agglomerates without compromising lattice integrity. The quantitative efficacy of this sequential post-treatment strategy is presented in Table 3.

**Figure 6:**
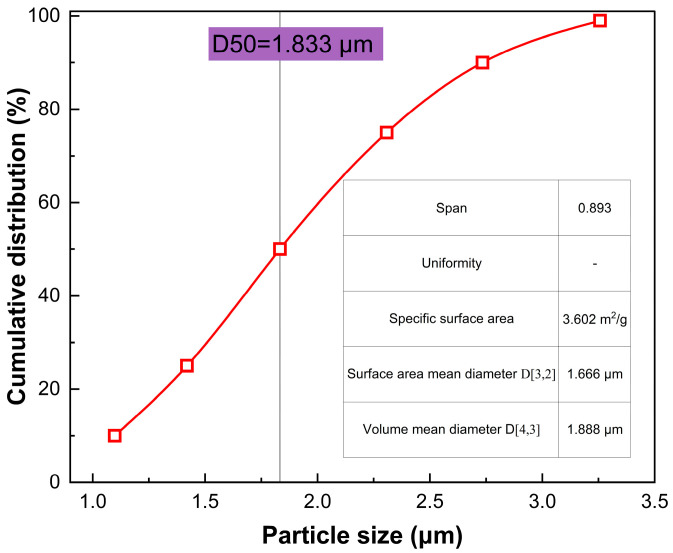
Distribution of particle sizes of silver powder subsequent to jet-milling deagglomeration.

Prior to jet-milling, the as-dried powder exhibits a D50 of 4.74 μm and a broad particle size distribution. Within the jet mill, a supersonic gas stream induces intense vortical turbulence. The resulting gas–solid two-phase flow subjects the particles to high-frequency self-collisions and aerodynamic shearing. This intense kinetic environment efficiently dismantles fragile soft agglomerates while strictly preserving the structural integrity of the primary crystallites. Post-deagglomeration, the D50 and D90 plummet to 1.83 μm and 2.73 μm, respectively, yielding a remarkably narrow distribution span of 0.89. The particle-size distribution after jet-milling deagglomeration is shown in Figure 6. This corroborates the attainment of a nearly perfect monodisperse normal distribution at the microscale. Ultimately, silver powders with such tailored metrics exhibit exceptional thixotropic flow and leveling characteristics, rigorously satisfying the stringent rheological demands of ultra-fine-line screen printing and zero-busbar (0BB) metallization for advanced TOPCon and HJT modules.

### 3.7. Phase Composition of the Optimized Ag Powder

**Figure 7:**
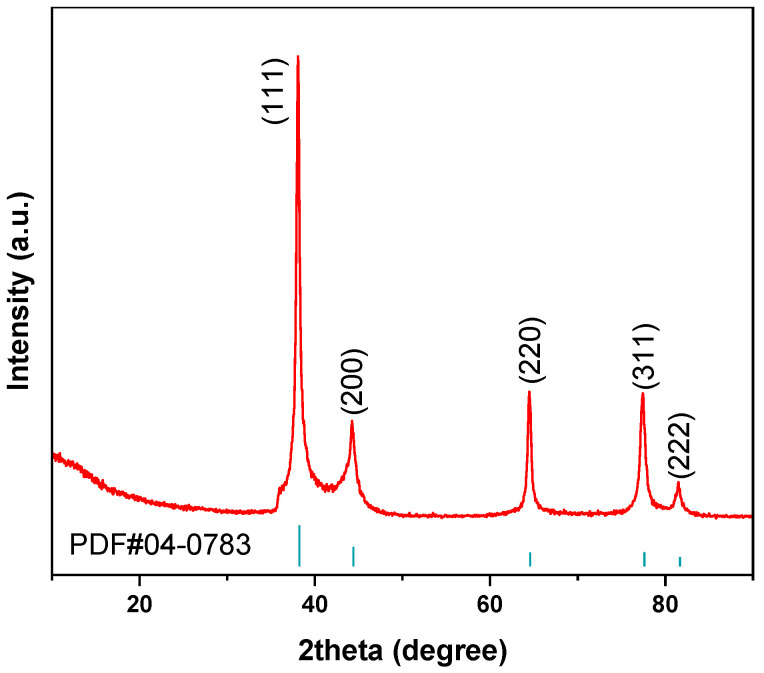
XRD pattern of the optimized final Ag powder.

XRD characterization was performed for the optimized final Ag powder to verify its crystalline phase composition and to examine whether crystalline Ag(I)-containing species remained after chemical reduction. As shown in Figure 7, the diffraction peaks at approximately 38.1°, 44.3°, 64.4°, 77.5°, and 81.5° can be indexed to the (111), (200), (220), (311), and (222) planes of face-centered cubic metallic Ag, respectively. These peaks agree well with the standard diffraction pattern of metallic Ag, PDF#04-0783. No obvious diffraction peaks corresponding to crystalline AgNO_3_ or Ag_2_O were detected. These results indicate that the main crystalline phase of the optimized powder is metallic Ag, and no XRD-detectable crystalline Ag(I)-containing salts or oxides were observed. However, because XRD is mainly sensitive to crystalline phases, trace ionic Ag(I), amorphous residues, or low-level impurities below the detection limit cannot be fully excluded by XRD alone.

## 4. Conclusions

In this study, we investigated the effects of reaction conditions, dispersant composition, and post-treatment on the liquid-phase reduction synthesis of ultrafine near-spherical Ag powders. The work focuses on powder synthesis and characterization, including particle-size distribution, SEM-observed morphology, tap density, specific surface area, and LOI. Through systematic process optimization and engineered post-treatment protocols, we successfully fabricated ultrafine, highly compact, and strictly monodisperse spherical silver particles with minimal organic residue. The principal conclusions are as follows:

(1) Ascorbic acid was definitively established as the optimal mild reducing agent. Guided by the LaMer model, an ideal thermodynamic and kinetic window was identified at 40 °C, a pH of 3.0, and an ascorbic acid/AgNO_3_ molar ratio of 1.3:2.0. These parameters effectively decouple burst nucleation from diffusion-controlled growth, unequivocally suppress secondary nucleation, and prevent the reverse dissolution of crystallographic facets, thereby laying a robust kinetic foundation for dense particle formation.
(2) A composite dispersant system comprising PVP and AG (1.0:2.0 mass ratio), integrated with a reverse rapid addition protocol, enables precise in-situ self-assembly at the solid–liquid interface. PVP executes targeted chemical passivation of high-energy facets to direct isotropic growth, while the bulky AG macromolecule constructs a resilient, hydrated steric barrier. This cooperative synergy decisively suppresses Ostwald ripening and shear-induced agglomeration, elevating the macroscopic tap density to an impressive 4.4 g/mL.
(3) Ethanol displacement and jet-milling treatment reduced apparent agglomeration and narrowed the particle-size distribution, giving a D50 of 1.83 μm and a span of 0.89. The LOI value below 1.0% at 538 °C for 30 min indicates relatively low total mass loss under the specified test condition. Because FTIR/IR or TGA analysis was not performed, the chemical nature and removal degree of residual surface-bound dispersants require further investigation. Ultimately, this exceptionally monodisperse powder demonstrates superior rheological compatibility, providing a definitive and scalable material solution for advanced ultra-fine-line and zero-busbar (0BB) metallization technologies in next-generation N-type high-efficiency solar cells.

## Figures and Tables

**Table 1:** Impact of various reducing agents on powder shape and particle dimensions.

Reducing Agent	Standard Redox Potential (V vs. SHE)	Kinetic Characteristics of Reduction	D50 (μm)	Distribution of Particle Size and Microscopic Morphology
Hydrazine hydrate	−1.16	Exceedingly swift response	1.10	Bimodal distortion; significant adhesion with uneven aggregation
Formaldehyde	−1.07	Moderately swift response	6.80	Bimodal distortion; mild particle agglomeration with heterogeneous dimensions
Glucose	−0.15	Delayed response	24.50	Extensive distribution range; significant coarsening induced by Ostwald ripening
Ascorbic acid	+0.39	Moderate pace	5.72	Continuous, symmetric unimodal distribution; dense and extremely monodisperse spherical particulates

D50 represents the median particle diameter, corresponding to the particle size at which 50% of the cumulative volume distribution is smaller than this value.

**Table 2:** Molecular-level synergistic mechanisms of the PVP and AG composite system.

Composition of Dispersant System (Mass Ratio)	Interfacial Chemical Passivation Effect	Steric Hindrance Shielding Effect	D50 (μm)	Tap Density (g/mL)	Assessment of Macroscopic Morphology
AG only	Weak	Strong	16.04	3.7	Significant coarsening; exposed facets promote atypical growth.
PVP only	Strong	Weak	9.84	3.6	Broadened distribution; monolayer protection vulnerable to shear-induced rupture.
PVP:AG = 1.0:1.0	Moderate	Moderate	5.74	4.0	Emerging synergistic effects; particle size distribution narrows.
PVP:AG = 1.0:2.0	Highly selective	Markedly enhanced	2.33	4.4	Improved composite stabilization; particles show relatively spherical morphology and increased tap density.

**Table 3:** Effect of sequential post-treatment on PSD, span, and physical dispersion state of Ag powder.

Preparation Phase of Silver Powder	D10 (μm)	D50 (μm)	D90 (μm)	Size Distribution Span	Evaluation of Physical Condition
Crude product obtained during liquid-phase reduction	1.95 ± 0.05	4.74 ± 0.12	16.07± 0.05	2.98 ± 0.09	Significant macroscopic soft aggregation; extensive spread with noticeable tailing.
Subsequent to ethanol displacement and desiccation	1.52 ± 0.04	2.46 ± 0.07	8.42± 0.26	2.80 ± 0.08	Minimised hard-cake aggregation; fine-scale dispersion necessitates enhancement.
Following jet-milling vortex deagglomeration	1.09 ± 0.03	1.83 ± 0.05	2.73± 0.08	0.89 ± 0.03	Achieved a highly symmetric monodisperse distribution; specific surface area completely released.

D10, D50, and D90 represent the particle diameters at cumulative volume fractions of 10%, 50%, and 90%, respectively. Span = (D90 − D10)/D50.

## Data Availability

The original contributions presented in this study are included in the article/Supplementary Material. Further inquiries can be directed to the corresponding author.

## Supplementary Materials

The following supporting information can be downloaded at: https://www.mdpi.com/article/10.3390/ma19102010/s1.

## References

[1] Zeng Y., Peng C.-W., Hong W., Wang S., Yu C., Zou S., Su X. (2022). Review on metallization approaches for high-efficiency silicon heterojunction solar cells. Trans. Tianjin Univ..

[2] Balaji N., Raval M.C., Saravanan S., Nayeripour M. (2020). Review on Metallization in Crystalline Silicon Solar Cells. Solar Cells.

[3] Tepner S., Wengenmeyr N., Linse M., Lorenz A., Pospischil M., Clement F. (2020). The link between Ag-paste rheology and screen-printed solar cell metallization. Adv. Mater. Technol..

[4] Yüce C., König M., Willenbacher N. (2018). Rheology and screen-printing performance of model silver pastes for metallization of Si-solar cells. Coatings.

[5] Thibert S., Jourdan J., Bechevet B., Chaussy D., Reverdy-Bruas N., Beneventi D. (2014). Influence of silver paste rheology and screen parameters on the front side metallization of silicon solar cell. Mater. Sci. Semicond. Process..

[6] Tsai J.-T., Lin S.-T. (2013). Silver powder effectiveness and mechanism of silver paste on silicon solar cells. J. Alloys Compd..

[7] Wang H., Tai Y., Li R., Wang H., Bai J. (2016). Effect of the mass ratio of micron and submicron silver powder in the front electrode paste on the electrical performance of crystalline silicon solar cells. RSC Adv..

[8] Yu X., Sun H., Qian Z., Li W., Li W., Huang F., Li J., Gan G. (2024). Effect of silver powder microstructure on the performance of silver powder and front-side solar silver paste. Materials.

[9] Liu Z., Qi X., Wang H. (2012). Synthesis and characterization of spherical and mono-disperse micro-silver powder used for silicon solar cell electronic paste. Adv. Powder Technol..

[10] Li N., Li J., Wan X., Niu Y., Gu Y., Chen G., Ju S. (2023). Preparation of micro-size spherical silver particles and their application in conductive silver paste. Materials.

[11] Zhang X., Li G., Jiang S., Wu H., Wu X., Zhang Y., Dong P., Zhou Z. (2025). Nano-silver powder for photovoltaic silver paste: Synthesis, technical principles, and future perspectives. J. Alloys Compd..

[12] Zhao M., Tang G., Yang S., Fu S. (2023). Parametric study on conductive patterns by low-temperature sintering of micron silver ink. RSC Adv..

[13] Abou El-Nour K.M., Eftaiha A.A., Al-Warthan A., Ammar R. (2010). Synthesis and applications of silver nanoparticles. Arab. J. Chem..

[14] Iravani S., Korbekandi H., Mirmohammadi S.V., Zolfaghari B. (2014). Synthesis of silver nanoparticles: Chemical, physical and biological methods. Res. Pharm. Sci..

[15] Mirzaei A., Janghorban K., Hashemi B., Bonyani M., Leonardi S.G., Neri G. (2017). Characterization and optical studies of PVP-capped silver nanoparticles. J. Nanostructure Chem..

[16] Bastús N.G., Merkoçi F., Piella J., Puntes V. (2014). Synthesis of highly monodisperse citrate-stabilized silver nanoparticles of up to 200 nm: Kinetic control and catalytic properties. Chem. Mater..

[17] Bai X.-H., Li W., Du X.-S., Zhang P., Lin Z.-D. (2017). Synthesis of spherical silver particles with micro/nanostructures at room temperature. Compos. Commun..

[18] Li Y.-F., Gan W.-P., Zhou J., Lu Z.-Q., Yang C., Ge T.-T. (2015). Hydrothermal synthesis of silver nanoparticles in Arabic gum aqueous solutions. Trans. Nonferrous Met. Soc. China.

[19] Whitehead C.B., Özkar S., Finke R.G. (2021). LaMer’s 1950 model of particle formation: A review and critical analysis of its classical nucleation and fluctuation theory basis, of competing models and mechanisms for phase-changes and particle formation, and then of its application to silver halide, semiconductor, metal, and metal-oxide nanoparticles. Mater. Adv..

[20] LaMer V.K., Dinegar R.H. (1950). Theory, production and mechanism of formation of monodispersed hydrosols. J. Am. Chem. Soc..

[21] Whitehead C.B., Özkar S., Finke R.G. (2019). LaMer’s 1950 model for particle formation of instantaneous nucleation and diffusion-controlled growth: A historical look at the model’s origins, assumptions, equations, and underlying sulfur sol formation kinetics data. Chem. Mater..

[22] Mo L., Guo Z., Wang Z., Yang L., Fang Y., Xin Z., Li X., Chen Y., Cao M., Zhang Q. (2019). Nano-silver ink of high conductivity and low sintering temperature for paper electronics. Nanoscale Res. Lett..

[23] Mdluli P.S., Sosibo N.M., Mashazi P.N., Nyokong T., Tshikhudo R.T., Skepu A., Van Der Lingen E. (2011). Selective adsorption of PVP on the surface of silver nanoparticles: A molecular dynamics study. J. Mol. Struct..

[24] Murshid N., Kitaev V. (2014). Role of poly (vinylpyrrolidone)(PVP) and other sterically protecting polymers in selective stabilization of {111} and {100} facets in pentagonally twinned silver nanoparticles. Chem. Commun..

[25] Rónavári A., Bélteky P., Boka E., Zakupszky D., Igaz N., Szerencsés B., Pfeiffer I., Kónya Z., Kiricsi M. (2021). Polyvinyl-pyrrolidone-coated silver nanoparticles—The colloidal, chemical, and biological consequences of steric stabilization under biorelevant conditions. Int. J. Mol. Sci..

[26] Majhi S., Kumar A., Sharma S., Tripathi C.S.P., Guin D. (2024). Gum Arabic-mediated synthesis of silver nanoparticles for their applications as colorimetric and SERS-based detection of hydrogen peroxide. Anal. Sci..

[27] Venkatesham M., Ayodhya D., Madhusudhan A., Veerabhadram G. (2012). Synthesis of stable silver nanoparticles using gum acacia as reducing and stabilizing agent and study of its microbial properties: A novel green approach. Int. J. Green Nanotechnol..

[28] Humbatova S., Tapdiqov S.Z., Zeynalov N.A., Taghiyev D., Mammadova S. (2017). Synthesis and study of structure silver nanoparticles by polyethyleneglycol-gum arabic polymers. J. Nano Res..

[29] Al-Ansari M.M., Al-Dahmash N.D., Ranjitsingh A.J.A. (2021). Synthesis of silver nanoparticles using gum Arabic: Evaluation of its inhibitory action on Streptococcus mutans causing dental caries and endocarditis. J. Infect. Public Health.

[30] Sun H. (1998). COMPASS: An ab initio force-field optimized for condensed-phase applications overview with details on alkane and benzene compounds. J. Phys. Chem. B.

[31] Bunte S.W., Sun H. (2000). Molecular modeling of energetic materials: The parameterization and validation of nitrate esters in the COMPASS force field. J. Phys. Chem. B.

[32] Özmetin C., Çopur M., Yartasi A., Muhtar Kocakerim M. (1998). Kinetic investigation of reaction between metallic silver and nitric acid solutions in the range 7.22−14.44 M. Ind. Eng. Chem. Res..

[33] Sadrnezhaad S.A.E., Mozammel M. (2006). Kinetics of silver dissolution in nitric acid from Ag-Au_0.04_-Cu_0.10_ and Ag-Cu_0.23_ scraps. J. Mater. Sci. Technol..

[34] Kameoka Y., Pigford R.L., Fundamentals E.C. (1977). Absorption of nitrogen dioxide into water, sulfuric acid, sodium hydroxide, and alkaline sodium sulfite aqueous solutions. Ind. Eng. Chem. Fundam..

[35] Suchak N.J., Jethani K., Joshi J.B. (1990). Absorption of nitrogen oxides in alkaline solutions: Selective manufacture of sodium nitrite. Ind. Eng. Chem. Res..

[36] Li D., Xiao Z., Aftab T.B., Xu S. (2018). Flue gas denitration by wet oxidation absorption methods: Current status and development. Environ. Eng. Sci..

[37] Chen J., Huang S., Zhong S., Tan W., Zeng G., Weng W., Chi X. (2025). Scalable synthesis of submicron spherical silver powders mediated by sulfate ions: Dual-function protection mechanism for morphology control and simplified wastewater treatment. J. Clean. Prod..

[38] Xie S., Chen W., Yu M., Lu G., Li K., Zhao W. (2025). Preparation and sintering behavior of micron-sized spherical-like sliver particles based on seed-mediated method. Mater. Today Commun..

