# Supplementary material for: Scalable Synthesis of High-Density Ultrafine Spherical Silver Powders
Source: Materials (Basel). 2026 May 12;19(10):2010. doi: 10.3390/ma19102010 (PMC13209088; doi:10.3390/ma19102010)
Supplement: Supplementary file 1 [file materials-19-02010-s001.zip › materials-4283121-supplementary.pdf]

Supporting Information

Scalable Synthesis of High-Density Ultrafine Spherical Silver Powders

Xi He [1], Jiangyong Pei [2], Xiaocai He [3] and Ruidong Xu [1,*]

[1] State Key Laboratory of Complex Nonferrous Metal Resources Clean Utilization,Faculty of Metallurgical and Energy Engineering, Kunming University of Science and Technology, Kunming 650093, China; hexi1@stu.kust.edu.cn
[2] School of Metallurgy, Northeastern University, Shenyang 110819, China; peijiangyong@mails.neu.edu.cn
[3] National Key Laboratory of Nonferrous Metal Reinforced Metallurgy New Technology, Nonferrous Metals Research Institute Co., Ltd., Aluminum Corporation of China, Kunming 650021, China; xc_he269@chinalco.com.cn
* Correspondence: rdxupaper@aliyun.com

Table S1. Key experimental and engineering parameters for Ag powder synthesis.

Parameter	Laboratory scale	Pilot scale
Reactor nominal volume	2 L	20 L
Working volume	1.20 L	12.0 L
Reactor inner diameter	120 mm	280 mm
Operating liquid height	ca. 106 mm	ca. 195 mm
Reactor material	Jacketed glass	Jacketed stainless steel
Impeller type	Four-blade pitched-blade turbine	Four-blade pitched-blade turbine
Impeller diameter	45 mm	100 mm
Impeller position above bottom	25 mm	60 mm
Number of baffles	4	4
Baffle width	12 mm	25 mm
Stirring speed during addition	600 rpm	300 rpm
Stirring speed during ageing	400 rpm	220 rpm
Reaction temperature	40 °C	40 °C
Initial pH	3.0	3.0
$AgNO_3$ concentration	2.0 mol/L	2.0 mol/L
$AgNO_3$ solution volume	465 mL	4.65 L
Ascorbic acid concentration	0.82 mol/L	0.82 mol/L
Ascorbic acid/dispersant solution volume	735 mL	7.35 L
Ascorbic acid/$AgNO_3$ molar ratio	1.3:2.0	1.3:2.0
PVP:AG mass ratio	1.0:2.0	1.0:2.0
Total dispersant dosage	1.5 wt.% of theoretical Ag	1.5 wt.% of theoretical Ag
PVP dosage	0.5 wt.% of theoretical Ag	0.5 wt.% of theoretical Ag
AG dosage	1.0 wt.% of theoretical Ag	1.0 wt.% of theoretical Ag
Feeding tube/nozzle diameter	2 mm	4 mm
Feeding position	Near impeller zone	Near impeller zone
$AgNO_3$ feeding rate	100 mL/min	1.0 L/min
Addition time	ca. 4.7 min	ca. 4.7 min
Ageing time	30 min	30 min
Theoretical Ag yield	ca. 100 g	ca. 1.0 kg

Table S1. Key experimental and engineering parameters for Ag powder synthesis (continued).

Parameter	Laboratory scale	Pilot scale
Solid loading	ca. 83 g Ag/L	ca. 83 g Ag/L
Hot-water washing temperature	60 °C	60 °C
Water washing liquid-to-solid ratio	10 mL/g	10 mL/g
Number of water washing cycles	5	5
Washing endpoint	pH 6.5–7.0, conductivity <50 μS/cm	pH 6.5–7.0, conductivity <50 μS/cm
Ethanol washing liquid-to-solid ratio	5 mL/g	5 mL/g
Number of ethanol washing cycles	3	3
Drying condition	40 °C, −0.1 MPa, 12 h	40 °C, −0.1 MPa, 12 h
Jet-milling gas	N_2	N_2
Jet-milling inlet pressure	0.65 MPa	0.65 MPa
Feeding rate for jet milling	20 g/min	150 g/min
Classifier speed	3200 rpm	3200 rpm
Number of milling passes	1	1
Final classification	300 mesh	300 mesh

Table S2. Statistical Summary Key Powder Properties.

Sample / condition	n	D10 (μm)	D50 (μm)	D90 (μm)	Span	Tap density (g/mL)	SSA (m²/g)	LOI (%)
Optimized PVP/AG system before post-treatment	3	—	2.33 ± 0.06	—	—	4.40 ± 0.08	—	—
Crude product obtained during liquid-phase reduction	3	1.95 ± 0.05	4.74 ± 0.12	16.07 ± 0.43	2.98 ± 0.09	—	—	—
After ethanol displacement and drying	3	1.52 ± 0.04	2.46 ± 0.07	8.42 ± 0.26	2.80 ± 0.08	—	—	0.99 ± 0.03
After jet-milling deagglomeration	3	1.09 ± 0.03	1.83 ± 0.05	2.73 ± 0.08	0.89 ± 0.03	—	0.60 ± 0.03	0.98 ± 0.02
Kilogram-scale pilot powder	3	1.12 ± 0.04	1.90 ± 0.06	2.84 ± 0.09	0.91 ± 0.04	6.00 ± 0.12	0.60 ± 0.03	0.98 ± 0.03

Table S3. Batch-to-batch consistency and Ag material balance for kilogram-scale pilot production.

Parameter	Batch 1	Batch 2	Batch 3	Mean ± SD
$AgNO_3$ concentration (mol/L)	2.0	2.0	2.0	—
$AgNO_3$ solution volume (L)	4.65	4.65	4.65	—
Theoretical Ag input (g)	1003.2	1003.2	1003.2	1003.2
Final dry powder mass (g)	979.4	988.7	983.2	983.8 ± 4.7
Overall powder yield (%)	97.6	98.6	98.0	98.1 ± 0.5
LOI (%)	0.96	0.99	0.98	0.98 ± 0.02
LOI-corrected Ag mass in product (g)	970.0	978.9	973.6	974.2 ± 4.5
LOI-corrected Ag recovery (%)	96.7	97.6	97.0	97.1 ± 0.5
Ag in mother liquor (g)	6.4	7.9	7.0	7.1 ± 0.8
Ag in washing filtrates (g)	5.2	6.3	5.9	5.8 ± 0.6
Ag in equipment/filter residues (g)	13.1	9.8	13.0	12.0 ± 1.9
Ag mass-balance closure (%)	99.2	99.9	99.6	99.6 ± 0.3
D10 (μm)	1.09	1.15	1.12	1.12 ± 0.03
D50 (μm)	1.84	1.95	1.91	1.90 ± 0.06

Table S3. Batch-to-batch consistency and Ag material balance for kilogram-scale pilot production (continued).

Parameter	Batch 1	Batch 2	Batch 3	Mean ± SD
D90 (μm)	2.76	2.91	2.85	2.84 ± 0.08
Span	0.91	0.90	0.91	0.91 ± 0.01
Tap density (g/mL)	5.89	6.12	5.99	6.00 ± 0.12
Specific surface area (m^2/g)	0.58	0.62	0.60	0.60 ± 0.02

Table S4. Reproducibility of outlet NOx concentration and nitrogen balance during Ag dissolution.

Run	Average outlet NOx (mg/m^3)	Peak outlet NOx (mg/m^3)	NOx limit in GB 31573-2015 (mg/m^3)	N recovered as NO_2^-/NO_3^- (%)
1	143	176	200	90.6
2	156	186	200	94.1
3	154	181	200	92.5
Mean ± SD	151 ± 8	181 ± 5	200	92.4 ± 2.1

Table S5 Evaluation of thermodynamic and chemical parameters affecting the dissolution kinetics of bulk silver ingots in a nitric acid system.

Group	Nitric Acid Concentration (%)	Temperature (°C)	Dissolution Time (h)
**I. Influence of Temperature**			
1	30	40	> 8
2	30	50	> 8
3	30	60	5–7
4	30	70	3–4
5	30	80	3–4
**II. Influence of Acid Concentration**			
6	10	75	> 8
7	20	75	> 8
8	30	75	3–4
9	40	75	3–4
10	50	75	~ 3
11	60	75	~ 3
**III. Kinetic Monitoring (Time Series)**			
12	30	75	0.5
13	30	75	1.0
14	30	75	1.5
15	30	75	2.0
16	30	75	2.5
17	30	75	3.0
18	30	75	3.5

Dissolution temperature critically governs reaction kinetics, surface passivation behavior, and NOx emission profiles during silver ingot dissolution. Systematic evaluation across five temperature points (40, 50, 60, 70, and 80 °C) was performed using 100 g silver batches with 100 mL deionized water and 100 mL 30% HNO_3 under constant stirring. Experimental results are summarized in **Table S5**. At 40–50 °C, dissolution exceeded 8 h, rendering the process industrially impractical. At 60 °C and above, reaction kinetics accelerated significantly. However, at 80 °C, violent exothermic behavior induced uncontrollable NOx boil-over and solution splashing. The optimal temperature window of 70–80 °C stabilized dissolution time at 3–4 h, balancing efficiency with process safety. Subsequent experiments adopted 75 °C as the fixed dissolution temperature.

Nitric acid concentration directly modulates dissolution rate and surface passivation extent. Six HNO_3 mass fractions (10%, 20%, 30%, 40%, 50%, and 60%) were examined at 75 °C under otherwise

identical conditions (**Table S5**). At 10–20% HNO_3, dissolution was prohibitively slow (>8 h). Increasing acid strength to 30–40% significantly enhanced mass transfer, reducing reaction time to 3–4 h. Although 50% HNO_3 further shortened the time to ~3 h, concentrations of 60% induced deep surface passivation and hazardous reactor overflow without meaningful further time reduction. Consequently, 30–40% HNO_3 was identified as the optimal range, ensuring efficient dissolution while mitigating passivation and operational risks.

Reaction time optimization was conducted under fixed conditions (75 °C, 30% HNO_3) with time-resolved monitoring from 0.5 to 3.5 h (**Table S5**). Macroscopic dissolution rate exhibited gradual decay as bulk $AgNO_3$ concentration increased, shifting rate control from surface reaction to Ag^+ outward diffusion. To overcome this late-stage kinetic bottleneck during scale-up, mass transfer intensification strategies (e.g., increased stirring rate or mild temperature elevation near reaction completion) are recommended to thin the Nernst diffusion boundary layer and accelerate ionic transport.

In summary, the optimized dissolution protocol employs a constant temperature of 75 °C, an initial HNO_3 concentration of 30–40%, and controlled mass transfer enhancement to achieve complete dissolution of 100 g silver ingots within 3–4 h. This methodology effectively circumvents deep surface passivation, ensures high precursor yield and purity, and establishes a stable foundation for green, scalable silver nitrate production. Nevertheless, the intrinsic chemistry of the Ag–HNO_3 system inevitably generates substantial NOx emissions. The subsequent section addresses the engineering of a dedicated tail-gas absorption and conversion system to eliminate this environmental liability.

Table S6 Real-time monitoring data of nitrogen oxides (NO_x) emission concentrations during the silver ingot dissolution process over time.

Sample No.	Sampling time (min)	NO_x Concentration (mg/m^3)
1	15	5124.6
2	30	5241.4
3	45	4986.3
4	60	5002.6
5	75	4783.1
6	90	4434.5
7	105	4025.9
8	120	3374.8
9	135	3013.4
10	150	2497.6
11	165	1547.6
12	180	842.3
13	210	300.7
14	240	195.9
15	270	100.8
16	300	69.5

The dissolution of silver ingots in nitric acid inevitably generates concentrated nitrogen oxide (NO_x) off-gases, posing significant environmental and operational hazards if directly vented. To mitigate this, an alkaline spray absorption system was implemented for deep tail-gas purification. Specifically, NOx exhaust is conveyed under negative pressure via a sealed manifold into a spray tower, where it undergoes gas–liquid mass transfer and neutralization with a circulating 3 mol/L sodium hydroxide (NaOH) solution. Achieving stable, reagent-efficient absorption, however, requires precise characterization of the dynamic NO_x release profile throughout the dissolution process.

Accordingly, real-time NO_x emission monitoring was conducted on an existing 30 kg per batch pilot-scale silver dissolution line over a complete 5-hour reaction cycle. Given the extensive solid–liquid interfacial area and vigorous initial reaction kinetics, conditions favoring a concentrated burst of NO_x, a high-frequency sampling interval of 15 minutes was adopted during the first 3 hours. As the reaction rate decayed with progressive acid consumption, NO_x generation stabilized, and the sampling frequency was reduced to 30 minutes for the final 2 hours.

This time-resolved, gradient sampling strategy accurately captures the concentration evolution of NO_x across the entire reaction lifecycle, elucidating the dynamic emission characteristics essential for adaptive spray-volume regulation. The corresponding real-time monitoring data and

concentration-time profiles are provided in **Table S6** and **Figure S2**.

Table S7. Detection results of fluorescence spectra of solid particles.

Element	Na	Mg	K	Cl	Si	Ag	Cl
Content (%)	17.000	0.030	0.020	0.009	0.005	0.004	0.002

Table S8. Influence of reaction temperature on the microstructure and physicochemical properties of silver powder.

No.	Sample	Tap Density (g/mL)	Weight Loss at 538 °C(%)	D50	D90	Morphology
1	35°C	4.1	1.07	6.00	12.82	Spherical
2	40°C	4.2	1.05	5.62	12.09	Spherical
3	45°C	4.2	1.16	3.3	7.4	Near-spherical
4	50°C	4.0	0.75	5.59	12.04	Near-spherical

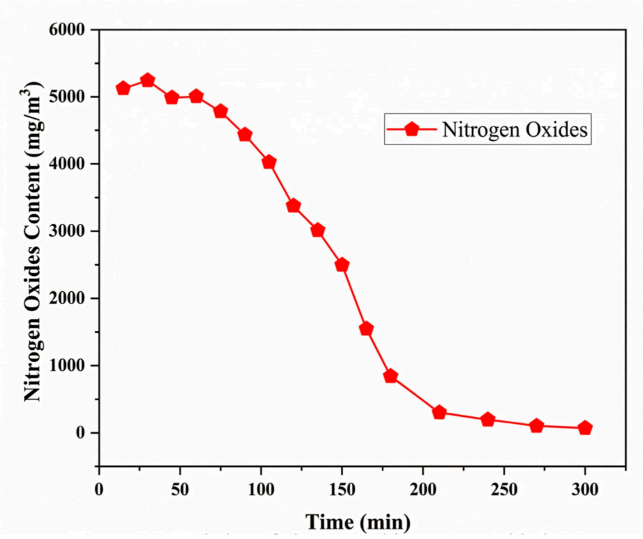

Figure S1. Variation of nitrogen oxides content with time.

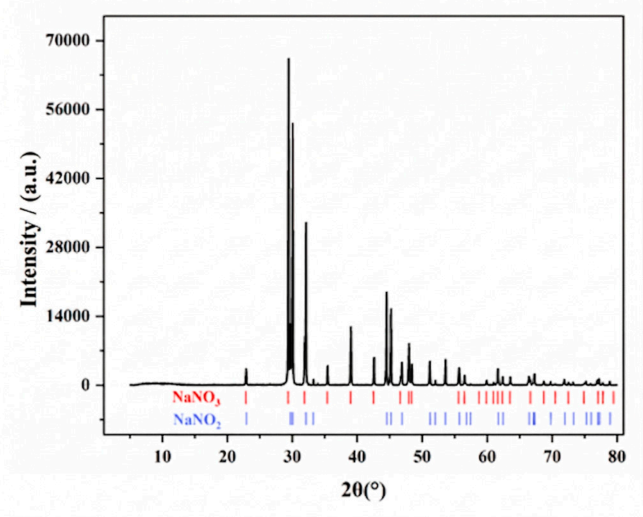

Figure S2. XRD pattern of evaporation.

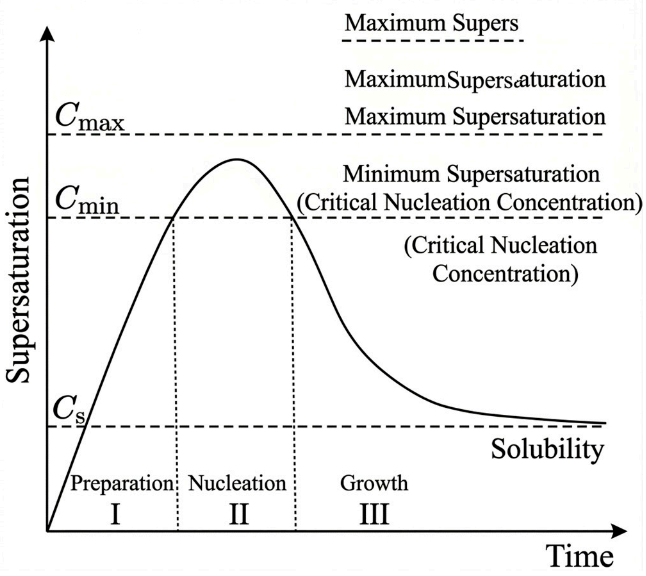

Figure S3. The Nucleation and growth mechanism diagram of the LaMer model.
